# Massive aspiration syndrome: a possible indication for “emergent” veno-venous extracorporeal membrane oxygenation?: a case report

**DOI:** 10.1186/s13256-021-03050-7

**Published:** 2021-10-08

**Authors:** Emiliano Gamberini, Venerino Poletti, Emanuele Russo, Alessandro Circelli, Marco Benni, Giovanni Scognamiglio, Domenico Pietro Santonastaso, Costanza Martino, Linda Domenichini, Romina Biondi, Giorgia Bastoni, Etrusca Brogi, Luca Ansaloni, Federico Coccolini, Paola Fugazzola, Martina Spiga, Vanni Agnoletti

**Affiliations:** 1 Anaesthesia and Intensive Care Department, Major Trauma Centre “Maurizio Bufalini” Hospital, 47251 Cesena, Italy; 2 Chest and Respiratory Diseases Division, Interventional Pneumology Department, Morgagni-Pierantoni Hospital, 47121 Forlì, Italy; 3 Emergency, General and Trauma Surgery Department, Level-1 Trauma Centre “Maurizio Bufalini” Hospital, 47251 Cesena, Italy; 4 Anaesthesia and Intensive Care Department, Level-1 Pisa University Hospital, Pisa, Italy; 5 Unit of General Surgery, “San Matteo” Foundation Hospital, Pavia University, Pavia, Italy; 6 Emergency, General and Trauma Surgery Department, Level-1 Pisa University Hospital, Pisa, Italy

**Keywords:** ECMO, Massive aspiration, Shock, ICU

## Abstract

**Background:**

Veno-venous extracorporeal membrane oxygenation (VV-ECMO) is usually performed in cases of severe respiratory failure in which conventional and advanced mechanical ventilation strategies are ineffective in achieving true lung-protective ventilation, thus triggering ventilatory-induced lung injury. If circulatory failure coexists, veno-arterial ECMO (VA-ECMO) may be preferred over VV-ECMO because of its potential for circulatory support. In VA-ECMO, the respiratory contribution is less effective and the complication rate is higher than in the VV configuration.

**Case presentation:**

The authors present a case in which VV-ECMO was performed in an emergency setting to treat a 68-year-old White male patient who experienced acute respiratory failure after massive aspiration. Despite intubation and intensive care unit admission, multiple organ failure occurred suddenly, thus prompting referral to a level-1 trauma center with an ECMO facility. The patient’s condition slowly improved with VV-ECMO support along with standard treatment for hemodynamic impairment. VV-ECMO was discontinued on day 8. The patient was extubated on day 14 and discharged home fully recovered 34 days after the event.

**Conclusions:**

Attention was focused on the decision to initiate VV-ECMO support even in the presence of severe hemodynamic derangement, although VA-ECMO could have provided better hemodynamic support but less effective respiratory support.

## Background

Veno-venous extracorporeal membrane oxygenation (VV-ECMO) is a thoroughly studied procedure for patients experiencing refractory severe hypoxemic respiratory failure [1]. Blood is withdrawn from the venous system into an extracorporeal circuit by a mechanical pump before entering an oxygenator and subsequently returned to the venous system. Although ECMO has been used to treat severe acute respiratory distress syndrome (ARDS) since 1972, results have been discouraging [2]. However, technological advances and the 2009 H1N1 influenza pandemic have provided a new perspective for VV-ECMO in the treatment of refractory severe respiratory failure [3]. In the past decade, new, highly specialized ECMO centers have been opened around the world to treat patients experiencing acute respiratory failure, building up hospital care networks following “a hub-and-spoke” model [4].

Massive aspiration is a potentially lethal complication that occurs in patients with decreased consciousness as a result of neurological impairment or during anesthesia procedures [5]. In cases of massive aspiration of gastric contents, a huge inflammatory response is triggered, leading to ARDS. In more severe cases, cardiopulmonary disturbances may coexist.

In this case, VV-ECMO was performed in an emergent setting of massive aspiration-related ARDS complicated with multiple organ failure (MOF). The decision to perform emergent VV-ECMO, instead of another ECMO configuration suitable for ensuring even circulatory support and more frequently used in this setting, was taken to maximize respiratory support.

## Case presentation

A 69-year-old White man, having previously undergone right hemicolectomy for colon cancer and with no chronic drug use or any other comorbidities, was admitted to the emergency department of a level-2 hospital for bowel obstruction and was immediately referred to the operating theater to undergo laparoscopic viscerolysis. The postoperative course was complicated on the sixth postoperative day by obstructive cholangitis with jaundice in the presence of bile sand and suspected choledocholithiasis; accordingly, antibiotics were prescribed, and endoscopic retrograde cholangiopancreatography (ERCP) was scheduled to be performed a few days later. Nine days after surgery, he was in good clinical condition. ERCP was performed using a conscious sedation technique; however, during the procedure, the patient suddenly experienced hypoxemia, tachycardia, and hypotension. Because aspiration of gastric contents was diagnosed, tracheal intubation was performed immediately, fluid resuscitation and continuous infusion of norepinephrine was initiated, and the patient was promptly transferred to the intensive care unit (ICU). For the sudden onset of severe ARDS, the patient was sedated and paralyzed to start lung-protective ventilation (LPV). A few hours after ICU admission, the patient experienced further worsening of MOF, and ICU medical staff decided to refer the patient to a level-1 hospital with an ECMO facility. Approximately 6 hours after massive aspiration, the patient was admitted to the authors’ level-1 trauma center hospital, and the ECMO team provided a consultation service in accordance with institutional criteria.

**Fig. 1:**
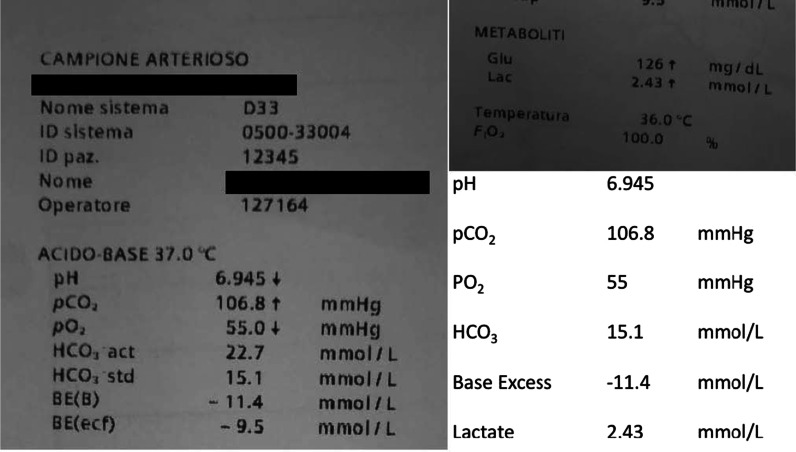
First arterial blood gas test on admission to the level-1 hospital

The patient arrived at the first evaluation with the ECMO team having undergone maximal medical treatment including deep sedation with neuromuscular blockers, mechanical ventilation over the limits of LPV [6], and continuous infusion of vasoactive and inotropic drugs to support circulation. Blood gas analysis revealed a pH of 6.9, a base excess of −11.4 mmol/L, a partial pressure of oxygen of 51.3 mmol/L notwithstanding a fraction of inspired oxygen (FiO_2_) of 100%, and a positive end-expiratory pressure (PEEP) of 10 cmH_2_O (Fig. 1). Pulmonary compliance was only 21 mL/mbar.

Systolic blood pressure fell to as low as 60 mmHg, and rapid ultrasound in shock (RUSH) [7] revealed bilateral white lung and global systolic dysfunction, as described in the clinical documentation.

## Therapeutic interventions

A sodium bicarbonate bolus was administered (consequent hypercarbia was not considered to be a contraindication because ECMO is highly effective in removing carbon dioxide regardless of configuration), with rapid improvement in mean arterial pressure (MAP) and cardiac systolic function on RUSH. Considering the severity of the respiratory failure (PaO_2_/FiO_2_, 0.55), the ECMO team decided to initiate ECMO in the veno-venous femoro-jugular configuration (drainage cannula, 23 French/55 cm in right femoral vein; return cannula, 19 French/14 cm in right internal jugular vein, Maquet Cardiohelp with the Quadrox-i membrane oxygenator, Getinge, Getinge, Sweden), even in the presence of hypotension on vasopressors and inotropes (epinephrine, 0.24 μg/kg/minute; norepinephrine, 0.57 μg/kg/minute; and dobutamine, 5.9 μg/kg/minute), considering the relatively low serum lactate levels (2.43 mmol/L).

ECMO support was initiated using a blood flow of 3.0 L/minute and a gas flow of 3 L/minute. Two units of red blood cells were administered, with a hemoglobin level > 10 mg/dL using central venous oxygen saturation (ScvO_2_) guidance to maintain a suitable ECMO preload.

During cannulation, a heparin bolus (5000 IU) was administered, and continuous infusion was started with a target activated partial thromboplastin time ratio of 1.5. Blood samples were drawn twice per day for thromboelastometric analysis. Continuous infusion of ketamine, sufentanil, and cisatracurium was chosen to manage sedation and paralysis to mitigate possible detrimental hemodynamic effects inherent in this strategy. LPV was set to 4 mL/kg ideal body weight, and tidal volume and respiratory rate to achieve pH > 7.2. PEEP was derived from the driving pressure and stress index [6].

A pulmonary artery catheter (PAC) was placed via the left subclavian vein for advanced invasive hemodynamic monitoring, indicating hypotensive hyperdynamic shock (MAP < 65 mmHg; heart rate > 120 beats/minute; cardiac index > 4.5 L/minute/m^2^, and systemic vascular resistance < 800 dyn·s/cm^5^/m^2^).

Intravenous fluids, dobutamine, and norepinephrine were adjusted under PAC guidance to reach the target in the lower range of normal. Continuous infusion of low-dose hydrocortisone was also initiated to maximize the effect of amines.

Stage 3 acute kidney injury developed at this time, and renal replacement therapy (RRT) was started in isovolemic continuous veno-venous hemodialysis and filtration mode. An in-line cytokine absorber helped to manage the “cytokine storm” and hyperinflammation.

**Fig. 2:**
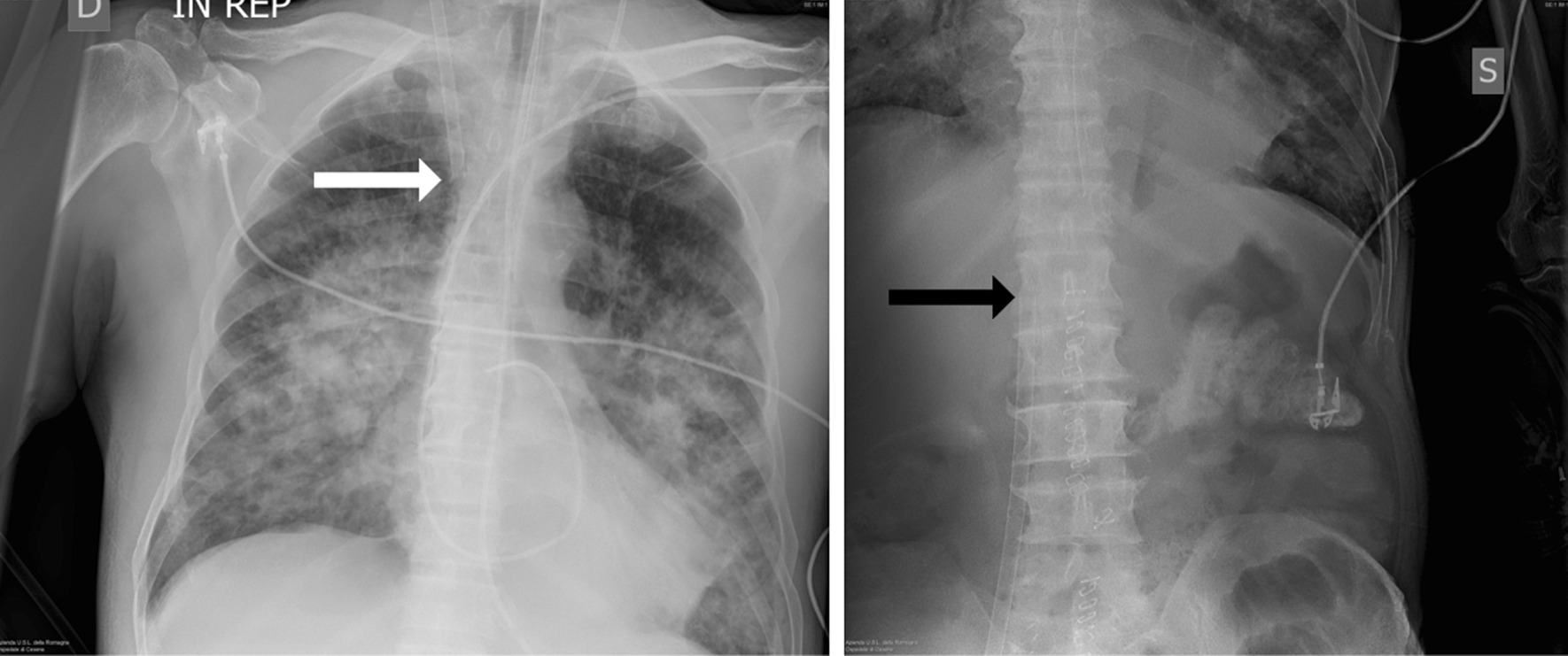
X-ray control for placement of cannulas for veno-venous extracorporeal membrane oxygenation. White arrow pointing the tip of the return cannula in superior vena cava while black arrow pointing the tip of the drainage cannula in inferior vena cava

**Fig. 3:**
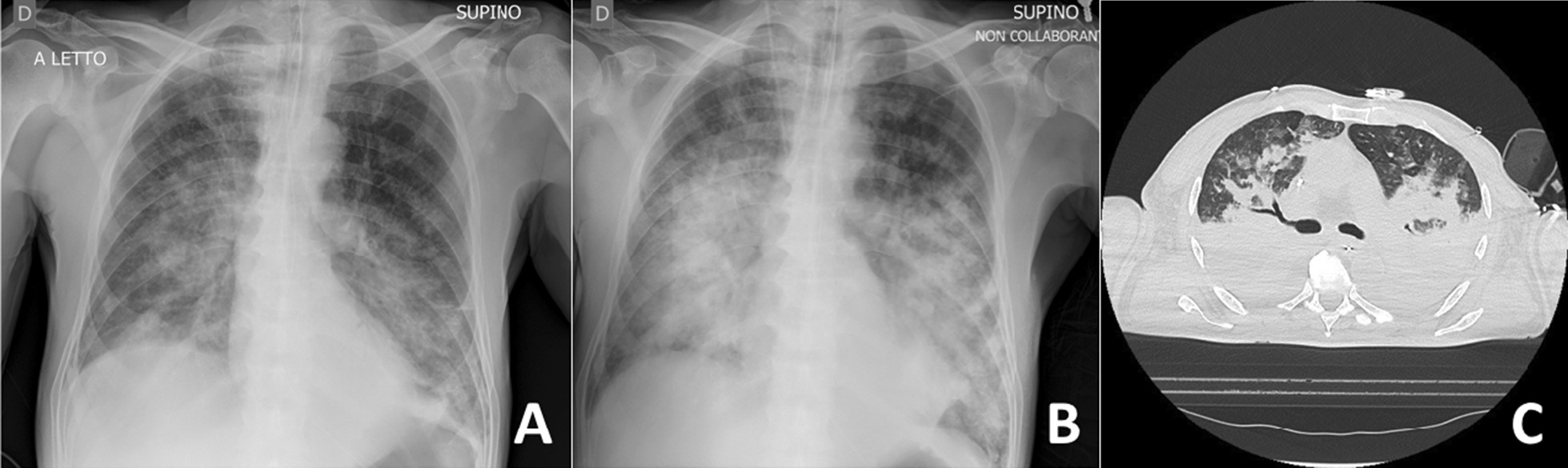
**A** Chest X-ray performed immediately after massive aspiration. **B** Chest X-ray 4 hours later in a level-2 hospital intensive care unit. **C** Lung computed tomography after initiation of veno-venous extracorporeal membrane oxygenation and clinical improvement in the level-1 hospital

**Fig. 4:**
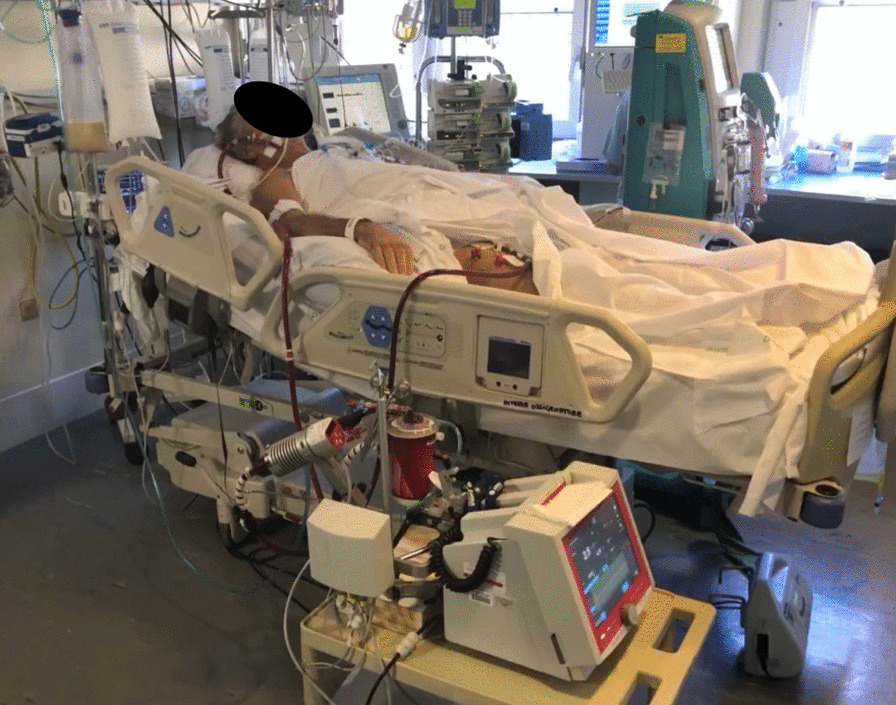
Extracorporeal centrifugal pump with membrane gas exchanger at the bedside

After clinical stabilization, chest computed tomography (CT) revealed posterior bilateral lung consolidation and diffuse ground-glass opacities, confirming severe ARDS (Figs. 2, 3). The ECMO team decided not to pronate the patient due to the good clinical response to treatment, although there was a persistent need for vasopressors and inotropes to achieve adequate MAP (Fig. 4).

Vasoactive and inotropic drugs, as well as neuromuscular blockers, were discontinued approximately 3 days after the event, with progressive de-titration guided by continuous cardiac output monitoring. Analgosedation was discontinued on day 6, a wake-up test was performed, and the patient promptly regained consciousness and pressure-support-assisted ventilation was set up. ECMO support was finally discontinued after a weaning trial on day 8, and the patient was extubated on day 14. Renal failure progressively returned to normal levels, and RRT was discontinued on day 20.

## Follow-up and outcome

The patient was transferred to the surgical ward on day 25 and was discharged on day 34 after full recovery. The patient has now been scheduled to undergo elective laparoscopic cholecystectomy.

## Discussion

The development of respiratory failure in patients with ARDS is usually progressive, and ECMO criteria are usually met a few days after the onset of the illness. When mechanical ventilation settings reach harmful thresholds in an attempt to ensure vital gas exchange, VV-ECMO must be considered to set a true LPV strategy. Usually, VV-ECMO has no emergent indications and is performed in accordance with strict selection criteria, and only after other treatments [6, 8]. VA-ECMO should be considered in cases of coexistent hemodynamic dearangement to support circulation when cardiac function remains severely depressed despite specific treatments such as inotropes  or other less invasive cardiac mechanical supports. In fact, in this configuration, the pump is capable of adding oxygenated blood flow in the descending aorta; however, physiopathological changes induced by VA-ECMO could worsen the positive effect on gas exchange by increasing cardiac afterload and, consequently, increasing pulmonary capillary pressure, adding a cardiogenic component to the already existing pulmonary edema. In addition, VA-ECMO is more difficult to manage than VV-ECMO.

Veno-arteriovenous (VAV)-ECMO can be considered an ideal solution for cardiovascular impairment associated with severe respiratory failure; however, it is significantly more complex and invasive, and likely less to be performed in an emergency setting [9]. To our knowledge, three cases of massive aspiration have been published. In two, VV-ECMO was performed to support respiratory function. Unfortunately, ECMO was not available at the time of the first reported case, in which the patient was discharged 1 year after hospital admission for treatment of sequelae after surviving massive aspiration [5, 9, 10].

In the present case of massive aspiration, the severity of respiratory failure fulfilled the criteria for VV-ECMO within a few hours. Prompt and aggressive correction of acidosis led to significant hemodynamic improvement. The associated inflammatory shock was transient, while pulmonary complications persisted for a longer time.

## Conclusions

Massive aspiration leading to ARDS and hemodynamic impairment could be a possible indication for “emergent” application of VV-ECMO as the first-line extracorporeal approach together with “traditional” circulatory resuscitation. VA-ECMO could be an option if severe circulatory failure coexists; however, attention must be devoted to the detrimental effect of this configuration on left heart function. VAV-ECMO may not be suitable for emergency settings; however, it can be considered if circulatory function does not improve after gas exchange correction using VV-ECMO or in case of persistent life-threatening respiratory failure after VA-ECMO initiation.

## Data Availability

Data sharing is not applicable to this article as no datasets were generated or analyzed during the current study.

## Abbreviations

VV-ECMO: Veno-venous extracorporeal membrane oxygenation
LPV: Lung-protective ventilation
VA-ECMO: Veno-arterial extracorporeal membrane oxygenation
VAV-ECMO: Veno-arteriovenous extracorporeal membrane oxygenation
MOF: Multiple organ failure
ARDS: Acute respiratory distress syndrome
ERCP: Endoscopic retrograde cholangiopancreatography
FiO_2_: Fraction of inspired oxygen
PEEP: Positive end expiratory pressure
PAC: Pulmonary artery catheter
RRT: Renal replacement therapy
CT: Computed tomography
